# Lateral drill holes decrease strength of the femur: an observational study using finite element and experimental analyses

**DOI:** 10.1186/1749-799X-8-29

**Published:** 2013-08-30

**Authors:** Melanie J Fox, Jennie M Scarvell, Paul N Smith, Shankar Kalyanasundaram, Zbigniew H Stachurski

**Affiliations:** 1 Department of Engineering, Australian National University, Canberra, ACT 0200, Australia; 2 Faculty of Health, University of Canberra, Bruce, ACT 2617, Australia; 3 Trauma and Orthopaedic Research Unit, Canberra Hospital, Woden, ACT 2606, Australia; 4 Australian National University Medical School, Australian National University, Canberra, ACT 0200, Australia

**Keywords:** Femur fracture, Internal fracture fixation, Intramedullary fixation, Finite element analysis, Stress, Mechanical, Materials testing

## Abstract

**Background:**

Internal fixation of femoral fractures requires drilling holes through the cortical bone of the shaft of the femur. Intramedullary suction reduces the fat emboli produced by reaming and nailing femoral fractures but requires four suction portals to be drilled into the femoral shaft. This work investigated the effect of these additional holes on the strength of the femur.

**Methods:**

Finite element analysis (FEA) was used to calculate compression, tension and load limits which were then compared to the results from mechanical testing. Models of intact femora and fractured femora internally fixed with intramedullary nailing were generated. In addition, four suction portals, lateral, anterior and posterior, were modelled. Stresses were used to calculate safety factors and predict fatigue. Physical testing on synthetic femora was carried out on a universal mechanical testing machine.

**Results:**

The FEA model for stresses generated during walking showed tensile stresses in the lateral femur and compression stresses in the medial femur with a maximum sheer stress through the neck of the femur. The lateral suction portals produced tensile stresses up to over 300% greater than in the femur without suction portals. The anterior and posterior portals did not significantly increase stresses. The lateral suction portals had a safety factor of 0.7, while the anterior and posterior posts had safety factors of 2.4 times walking loads. Synthetic bone subjected to cyclical loading and load to failure showed similar results. On mechanical testing, all constructs failed at the neck of the femur.

**Conclusions:**

The anterior suction portals produced minimal increases in stress to loading so are the preferred site should a femur require such drill holes for suction or internal fixation.

## Background

The aim of this study was to analyse the effect of additional drilled suction holes on femur strength and fatigue life. The effects of the suction holes have been investigated through the analysis of the induced stresses around the sites of the holes in addition to the stress distribution throughout the femur. Intact, fractured and nailed, and nailed femora with suction portals were studied using FE modelling. From the analysis of the stress distribution, the optimal location for the suction portals is derived for the studied conditions. The results of FEA were compared with experimental testing using synthetic composite bone.

Fat embolism is a regular feature of internal fixation for long-bone fractures [1,2], though clinical appearance of symptoms is less frequent [3]. Reports of the incidence of clinical fat embolism syndrome in reamed femur and tibia fractures range from 0.9% [4] to 74% [5]; these reports vary depending on the criteria for diagnosis [6,7]. The volume of fat emboli and the clinical outcome of fat embolism syndrome can be affected by several factors, including the timing and techniques of surgical fixation [8]. Both reamed and unreamed techniques have demonstrated similar rates of fat embolism [9,10].

While both reamed and unreamed intramedullary nailing cause fat extravasation, a reamed internal fixation technique permits a larger nail to be inserted, stronger locking bolts and a more stable internal fixation system [11], consequently a minimally traumatic reaming system is sought. The reamer irrigator aspirator system (DePuy Synthes, West Chester, PA, USA) [12-14] is one such system designed to reduce intramedullary pressure and fat extravasation, distal venting of the femur is another [15]. The intramedullary suction system (ISS) [16-18] combines distal venting and suction with monitoring of intramedually pressure. However, ISS requires four portals to be drilled into the femur, two portals for application of suction and two portals for pressure monitoring. In animal tests, the intramedullary pressure was effectively reduced, intraoperative respiratory observations were improved and fat emboli were reduced when the ISS was used [16].

One concern associated with the use of ISS is the addition of four 5-mm portals in the femur in addition to the screw holes required for the intramedullary nailing system [16]. It is not known if the additional holes decrease the structural integrity of the bone, though studies of animals [19] and cadaveric specimens [20] suggest this may happen. Seeking to measure and model the stresses around these additional holes is, therefore, of significant interest and medical benefit. The finite element analysis (FEA) supported by experimental testing has shown that the intramedullary nail and cross screws have a load-sharing contribution [21,22], and the load accepted by the remaining cross screws is increased if one is removed. This warrants a comparison between an intact femur and a femur fixed by intramedullary nailing, both with and without the inclusion of suction portals.

## Methods

### Experimental materials

The bones used in the mechanical testing were synthetic femurs produced by Sawbones (Pacific Research Laboratories, Vashon, WA, USA). The synthetic bones were used as they provide identical geometry and material properties across samples that cannot be obtained if using natural bones.

**Figure 1:**
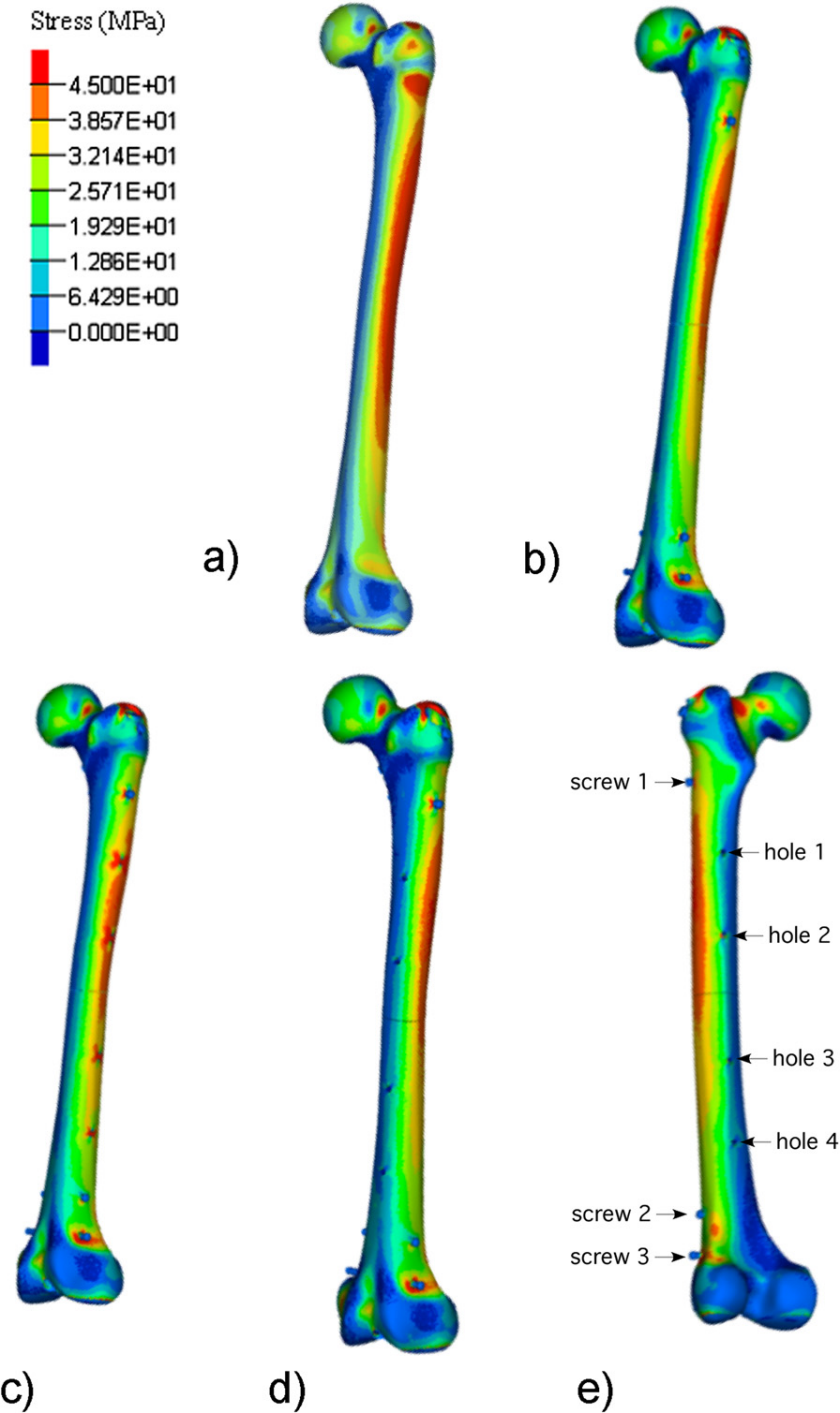
**Tensile stress contour plots.** For intact **(a)**, fractured and nailed **(b)** and fractured and nailed with 5-mm intramedullary suction portals *in situ***(c, d, e)**.

The intramedullary nailing system used for the model and testing was the Synthes Expert Lateral Femoral Nail (Synthes GmbH, Solothurn, Switzerland). The system treats femoral fractures effectively and is the preferred system for use by this hospital. The intramedullary nail used was the 420-mm Expert Lateral Femoral Nail with a 6.5-mm hip screw and three 5-mm locking screws (one proximal and two distal). Two ISS suction holes were placed proximal to the fracture, and two distal (Figure 1e). A model with medial suction holes was not investigated.

### Development of the finite element model

The geometry of the model was created in Solidworks (Dessault Systems, Providence, RI, USA). The finite element model of the composite femur was created by Papini et al. [23], later modified by Desmarais-Trepanier [24] and was made available on the internet through the BEL Repository managed by the Instituti Orthopedici Rizzoli, Bologna, Italy. However, in the finite element model of the femur, only the cortical bone has been included.

The Solidworks model was imported into Altair HyperMesh (Altair Engineering, Inc., Troy, MI, USA) to create the finite element mesh. The model was simplified in the same manner as Chen et al. [21] and Cheung et al. [22] by omitting the contribution of cancellous bone (as it contributes less than 1% to strength) and the longitudinal grooves in the intramedullary nail. The FEA mesh was refined until the resulting displacements converged between the models. Around the screw holes and the suction portals, the mesh was refined further until the stresses, rather than displacements, converged. The cortical femur bone was modelled using approximately 1,420,000 to 1,565,000 tetrahedral elements.

The FE models and analysis were generated using ABAQUS (Simulia, Dessault Systems) and Hyperworks (Altair) software packages. The programs were run on the supercomputing facilities at the Australian National University.

Seven constructs of the femur were modelled:

(1)  An intact femur.

(2)  A fractured, reamed and nailed femur (transverse midshaft fracture).

(3)  A fractured, reamed and nailed femur with four 5-mm suction portals positioned laterally.

(4)  A fractured, reamed and nailed femur with four 5-mm suction portals positioned anteriorly.

(5)  A fractured, reamed and nailed femur with four 5-mm suction portals positioned posteriorly.

(6)  A fractured, reamed and nailed femur with four enlarged (10 mm) suction portals positioned laterally.

(7)  A fractured, reamed and nailed femur with four enlarged (10 mm) suction portals positioned anteriorly.

First, a load protocol corresponding to stance phase of walking was modelled including load bearing through the proximal femoral head, muscle forces through the greater trochanter and ground reaction forces through the distal femoral condyles, with magnitude corresponding to 750 N bodyweight [25,26]. Assumptions were made with regard to the bone and nail being bonded throughout the FE analysis; the fracture surfaces were in contact, did not slide and did not penetrate each other. The effects of the suction portals were quantified through the use of factors of safety in addition to fatigue life predictions based on *S*-*N* data for human bones [27]. For both the walking and mechanical testing load cases, the distal end of the femur was fixed. At the heel strike phase of gait, the distal femur is effectively ‘locked’ in position at the knee joint, so this boundary condition is suitable.

Second, a load protocol corresponding to the maximum compressive load was modelled. The load was applied directly through the head of the femur to the condyles. This was used to make maximum failure load predictions for the seven constructs.

### Mechanical testing

Mechanical testing was conducted to obtain physical results and to compare the results with the FEA predictions. The tests were carried out using an Instron 4505 universal testing machine (Instron Pty Ltd., Norwood, MA, USA). To ensure that the load applied to the femur was representative of how the femur is loaded in the human body, two specialised fittings were made for the Instron mechanical testing machine. The top fitting was moulded using an epoxy resin impression to allow the proximal half of the femoral head to be encased, ensuring the load was applied at the centre of the head. The base fitting ensured that the femoral shaft was at an angle of 4°, and the alignment of the load was from the centre of the femoral head to the centre of the knee. To ensure that the system behaves as it does in the human body, contact conditions were established between the bone and the nail.

The synthetic femora were subjected to mechanical fatigue cycles to simulate the compressive aspect of walking and then followed by pure compression load to failure. Eighteen synthetic femora were used: three intact, five fractured, reamed and nailed, five with lateral suction portals and five with anterior suction portals present. The fatigue cycle comprised 1,000 compressive cycles, with the load cycling sinusoidally from 200 to 3,000 N. Each cycle took, on average, 3.6 seconds to complete, with the total time for each fatigue test being approximately 1 h.

In addition to those femurs subjected to fatigue testing, two femurs for each of the constructs were subjected to compression testing. For the compression tests, the femur was compressed at a rate of 1 mm/min until the bones failed. The corresponding load to failure was recorded. Fatigue and compression samples were examined prior to and following testing to observe the position of failure and any other changes in the features of the femora.

## Results

### Finite element computations

**Figure 2:**
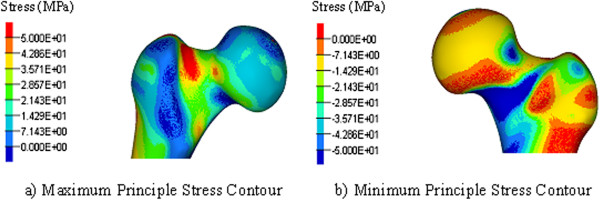
**Stress contours of the femoral head, demonstrating peak stress at the neck of the femur.** Maximum **(a)** and minimum **(b)**.

The standard walking condition results in a bending of the bone during stance phase. This generates tensile stresses in the lateral femur and compressive stresses in the medial femur, as shown for the first five constructs in Figure 1a,b,c,d,e. The maximum tensile stresses also occur at the femoral neck as shown in Figure 2. These stresses dominated those produced by the hip screw. At all locking screw holes, the maximum stresses occurred in the regions surrounding the screws.

Along the anterior and posterior femur, less stress is evident; hence, these are ideal for locating the suction holes. The placement of suction holes along the anterior or posterior surfaces produced little effect on the stresses as they are positioned on the neutral bending axis.

**Figure 3:**
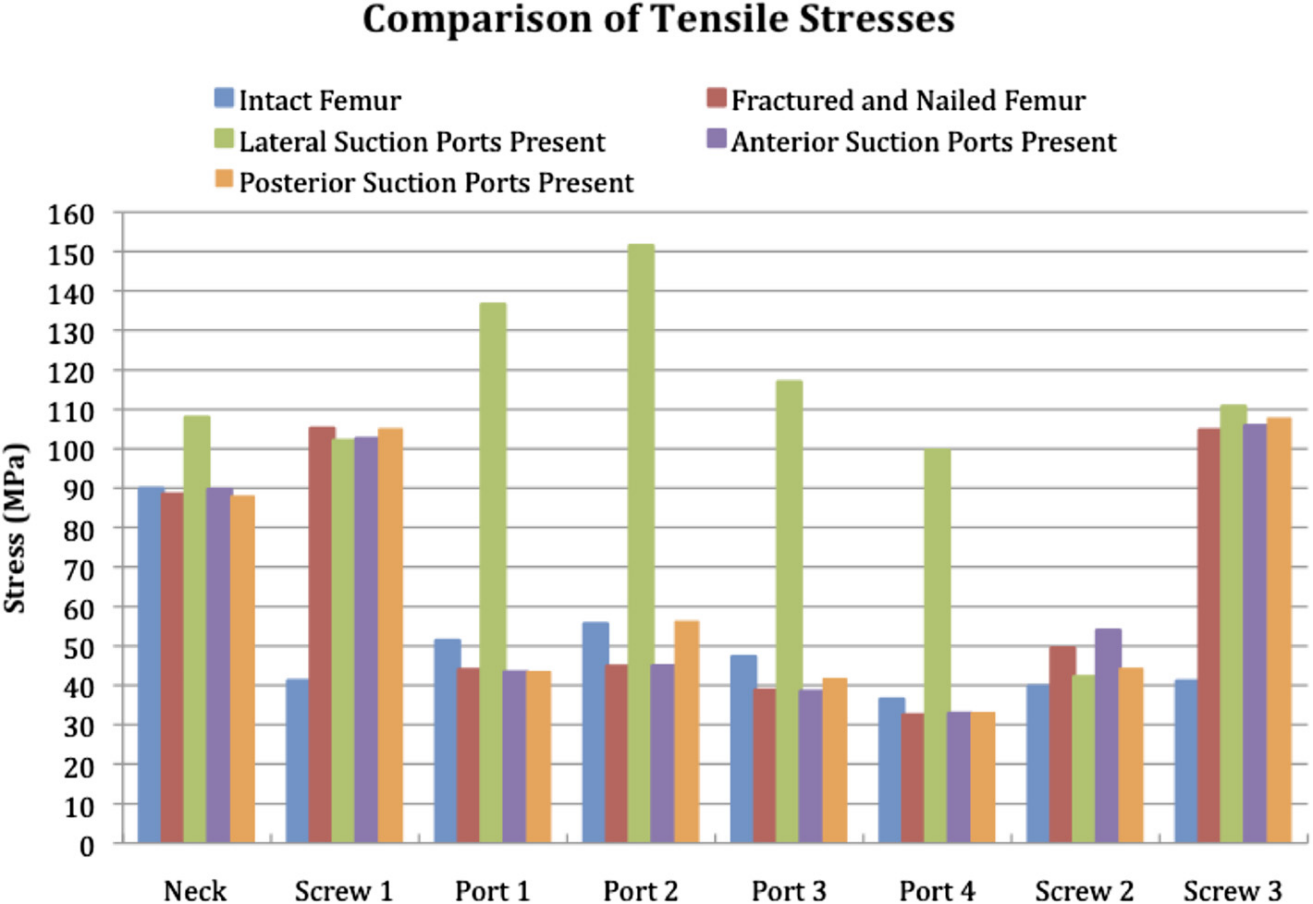
A comparison of maximum tensile stresses throughout the femur demonstrating tensile stress at the lateral portals.

**Figure 4:**
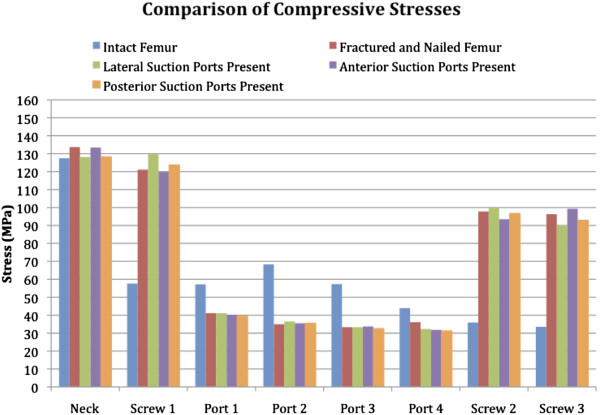
A comparison of maximum compressive stresses throughout the femur for the stance phase of walking.

A comparison of the tensile stresses for the five constructs is shown in Figure 3, and a similar chart for the compressive stresses is shown in Figure 4. The maximum tensile stress, significantly higher than other stresses, is seen in the femur with the portals positioned laterally. In the scenario with lateral suction portals, the failure is predicted to occur through the site of suction portal 2. In the case of laterally placed suction portals, the portals negatively affected the mechanical performance of the bone.

**Figure 5:**
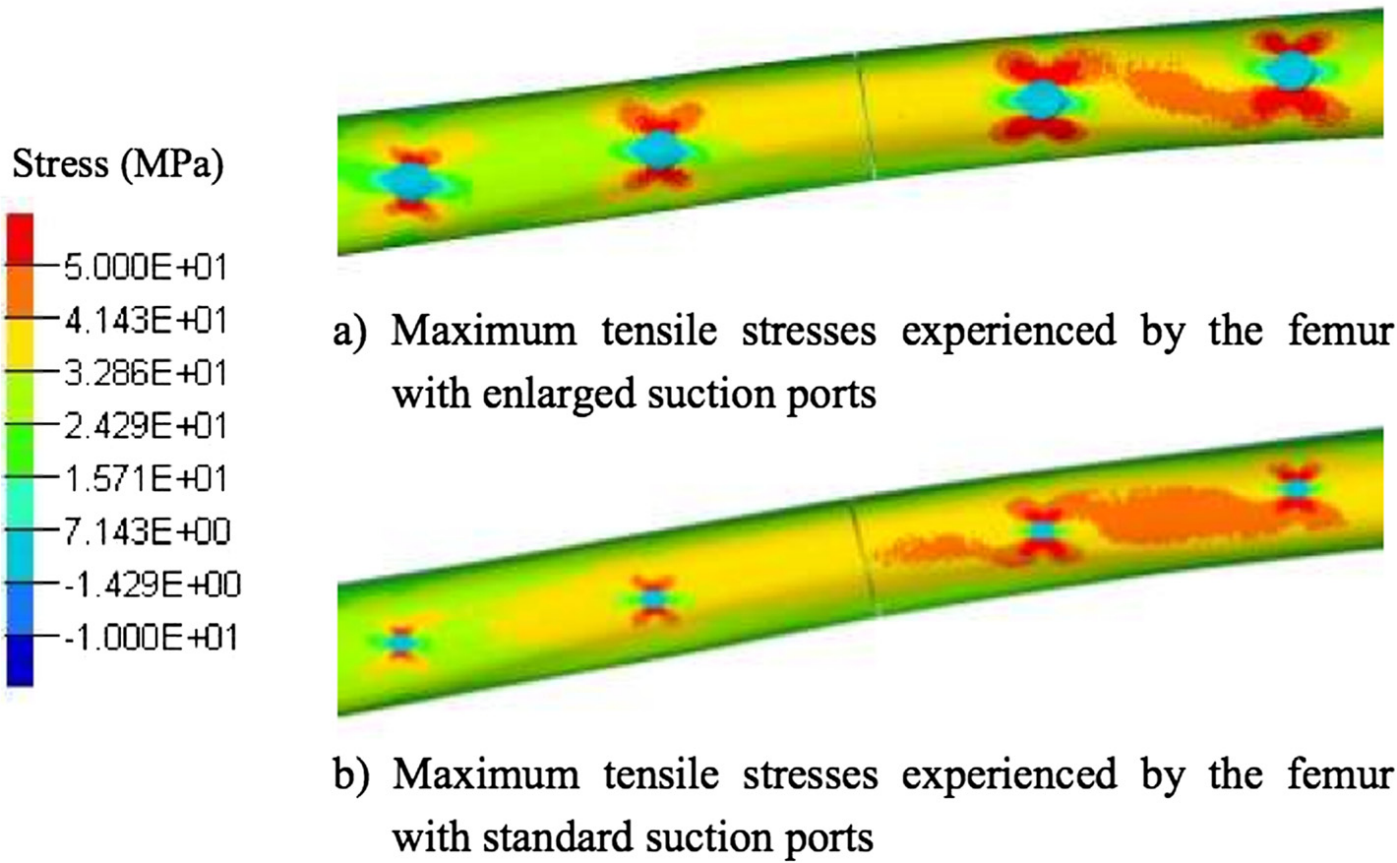
**Comparison of tensile stresses at different sizes of suction portals. (a)** 10 mm and **(b)** 5 mm.

Enlarging the size of the suction portals in the FEA model from 5 to 10 mm made no significant difference to the stresses experienced at any portals (Figure 5). Increasing the size of the lateral femur portals increased the tensile stresses by less than 7% (from 128 to 132 MPa); size increase on anterior suction portals had no effect. The compression stresses experienced at all suction portal sites were similar (mean 35 to 41 MPa) with no difference due to enlarged portals.

Prediction of the site within the femur at which the construct would fail always pointed to the femoral neck as the site of failure for all constructs (Figure 1). The high stresses in the trochanteric area are more significant for the mechanical testing load case as the applied load is more compressive than that applied for the walking load case.

### Safety factors for suction portal consideration

Under standard walking loads, the factor of safety for the intact femur was 1.9 times the walking load modelled; that of the fractured and nailed femur was 2.4. The limiting location for all of the constructs with suction portals was the site of suction portal 2. The safety factor for the lateral portals *in situ* was 0.7. The safety factor of less than 1 indicated that the femur would fail at this site, under this load. For anterior portals *in situ*, the safety factor was 2.4, and for posterior portals, the safety factor was 1.9. Consequently, it was only the lateral suction portals that reduced the safety factor, anterior portals did not.

### Mechanical testing

Under fatigue loading, none of the femora failed. No cracking or signs of failure were observed at any of the screw holes or suction portals. One sample with anterior suction portals showed some cracking near the neck of the femur.

The intact femora had the highest load at failure of 8,298 ± 86 N, and the smallest variation in the values obtained. The fractured and nailed femora failed at 7,772 ± 535 N. The femora with suction portals failed at lower compression loads: the lateral suction portal scenario failed at 6,537 ± 842 N, and the anterior suction portal scenario failed at 5,864 ± 1,085 N. The reaming of the bones caused some cracks around the proximal entry to form. Some of these cracks then propagated as a result of the stresses experienced when the intramedullary nail was inserted.

The pattern of failure was consistently through the proximal femur. The intact femora failed by transcervical fracture by midcervical shear. The fractured and intramedullary nailed femora failed by transcervical fracture. The femora with suction portals present (anterior, posterior or lateral) failed by simple pertrochanteric fracture. The fractures initiated at the proximal nail entry and propagated along the intertrochanteric line.

## Discussion

This study analysed the effect of suction portals drilled into the femoral shaft for intramedullary suction on femur strength and fatigue life using finite element analysis. The induced stresses around the sites of the holes were greatest in the lateral portals and least in the anterior portals. Enlarging the suction portals from 5 to 10 mm increased the tensile stresses by less than 7%. The experimental testing with fatigue cycles to simulate the compressive aspect of walking showed little damage, and pure compression load to failure demonstrated failure at the neck of femur in all test samples, with none failing at the suction portals.

**Figure 6:**
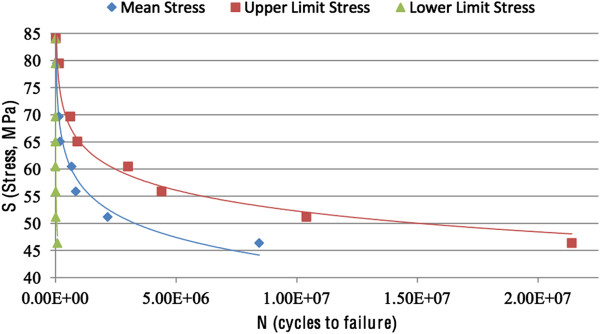
**Interpretation of data.** Interpretation of data for an *S*-*N* curve for the human femur ([27]) demonstrating the mean number of cycles to failure at particular stress amplitudes.

The *S*-*N* curve for the human femur was constructed utilizing the comprehensive set of data available in the work of Swanson et al. [27]. This type of testing subjects the samples to alternating tensile and compressive stresses, with the average stress over a complete cycle being zero. The data obtained was analysed in the 1970s without the use of a computer or modern data analysis tools, so equations describing the curve were not provided in the study. Figure 6 illustrates the data, graphed and analysed in Microsoft Excel to obtain a relationship between *S* and *N*. The mean number of cycles to failure at particular stress amplitudes was used to account for the range of ages and genders of the samples. For analysis, the mean curve was used for fatigue life predictions. It should be noted that the *S*-*N* relationships were for cadaveric bone, so the predictions are unlikely to hold for bone *in vivo* and only provide very conservative estimates.

Mathematical and finite element analysis of the femur has demonstrated that the lateral cortices of the femoral shaft are under tensile stresses, while the medial cortices are under compressive stresses [28,29]. This was also demonstrated in this study. However, our study also shows that the tensile stresses in the lateral femur with lateral suction portals drilled were beyond the safety limits of the femur in the stance phase of gait.

Our research shows that the anterior and posterior cortices of the femur are safer for portal drilling than the lateral cortices. The application of the four suction portals to the reamed and nailed femur appears to be within the safety tolerances for walking and does not provide a significantly earlier compression failure, provided that the portals are placed anteriorly. The laterally placed portals are not preferred as they provide the weakest construct in any of the testing protocols. This is the first time this has been demonstrated in FEA [22].

The point of failure for each of our tested scenarios was through the femoral neck, not through the portal sites or the screw holes. Despite evidence of high tensile stresses at these points, the stresses at the femoral neck proved limiting (Figure 2). While the intramedullary nailing system includes the hip screw, the hip screw did little to support the load compared to the distal locking screws. The load sparing effects of the nail and locking screws is described by Cheung et al. [22] and Chen et al. [21] who similarly used FEA validated by mechanical testing, but in a retrograde intramedullary nailing system. However, neither study went so far as to predict the failure points in the construct and so does not mention the neck of femur. Also, their strain measurement of the mechanical experiments did not include the neck of femur.

This paper does not extrapolate these findings to clinical decisions as extensive validation would be required before this was suggested. The use of suction portals in this model was based on the development of the intramedullary suction system [16,17]. This system employs two portals for suction and two portals for pressure monitoring so that while reaming, the pressures can be kept to limits that minimise the extravasation of fat. An alternative method developed to reduce fat embolism during reaming was the reamer-irrigator-aspirator system [30], which also cooled the bone during the reaming. Since then, its use for autograft and stem cell harvest has dominated the literature [30]. The ISS, however, provides real time information on the reaming pressures which are the main correlate with fat extravasation [16]. The disadvantage of the ISS is the requirement for four suction portals. It is essential that the portals are placed in the aspect of the femur least likely reduce the load capacity of the femur. Furthermore, for clinical decisions, this paper also makes no comment on the placement of drill holes required for plates and screws for fixation of diaphyseal fractures of the femur with compression or interlocking plates. Diaphyseal fractures are most commonly fixed with intramedullary nailing [31], but plates may be used for the metaphysis or periprosthetic fractures. In this study, much of the load was born by the intramedullary nail and locking screws, so extrapolation to plate fixation is impossible.

This work was limited by the constraints of using synthetic femora which, while controlling sample variability, were also prone to damage during sample preparation. For the intact femur, only one sample was subjected to fatigue testing as literature suggested that failure occurred only after 10 million cycles [32], and this number of cycles could not be completed in a suitable testing time frame. Under pure compression, the loads at failure demonstrated that there was variability in the maximum for all of the samples which were both reamed and nailed, possibly due to the damage to the proximal femur during the reaming and nailing processes. This resulted in only two of the three prepared femurs being tested. Additionally, while three femurs were prepared for the scenario with suction portals placed laterally, substantial damage occurred around the hole where the main nail was to be inserted, so only two of the femurs were tested. However, the consistent failure patterns suggest that the sample size was sufficient. This paper would have been strengthened by the application of validity testing regimes to the research method.

This work utilised an FEA model of more complexity and has greater numbers of elements involved in the analysis compared to the previous studies, in addition to a complete fracture of the femoral shaft and contact between the bodies being included. This was possible by the use of the supercomputing facility at the ANU. This model may be applied to other research questions around femoral loading in the future.

## Conclusions

This study has provided FEA data to support decisions over preferred placement of the suction portals used in the intramedullary suction system. In this study, two loading protocols were used, one mimicking a walking load, and the other mimicking a mechanical compression testing load. While the lateral portal placement showed the highest tensile stresses and did not meet safety requirements, the anterior and posterior portal placements were not significantly different in terms of tensile and compressive stresses. Likewise, increasing the size of the portals from 5 to 10 mm increased stresses less than 7%. Under the mechanical testing, all constructs failed at the neck of the femur, not at the suction portals. Future research on the placement, number and alignment of portals can then be used to inform clinical procedure with regard to the implementation of the suctioning system.

## Competing interests

No funding was received for the conduct of this research, but surgical implants were provided by Synthes Australia for use in the experiments. No funding was received by the authors from any commercial entity in reimbursements, salary, fees, stocks or shares for the conduct of this research.

## Authors’ contributions

MF carried out the finite element modelling, the testing of materials, conducted the analysis and wrote up the results. JS assisted in the preparation of the experiments, participated in the design of the study and drafted and revised the manuscript. PNS provided the original ideas, participated in the design of the study and provided consultancy and advice. SK participated in the design of the study and supervised the FEA. ZS participated in the design of the study, supervised the materials testing and edited and revised the manuscript. All authors read and approved the final manuscript.
